# Crystal structure analysis of [5-(4-meth­oxy­phen­yl)-2-methyl-2*H*-1,2,3-triazol-4-yl](thio­phen-2-yl)methanone

**DOI:** 10.1107/S2056989018010654

**Published:** 2018-07-31

**Authors:** Subhrajyoti Bhandary, Yarabhally R. Girish, Katharigatta N. Venugopala, Deepak Chopra

**Affiliations:** a Crystallography and Crystal Chemistry Laboratory, Department of Chemistry, Indian Institute of Science Education and Research Bhopal, Bhopal By-pass Road, Bhauri, Bhopal 462 066, Madhya Pradesh, India; b Department of Organic Chemistry, Indian Institute of Science, Bangalore 560 012, Karnataka, India; c Department of Biotechnology and Food Technology, Durban University of Technology, Durban 4001, South Africa

**Keywords:** crystal structure, 1,2,3-triazole, hydrogen bonding, mol­ecular electrostatic potential, MESP, fingerprint plot

## Abstract

The title compound, C_15_H_13_N_3_O_2_S, crystallizes in the monoclinic space group *P*2_1_/*n* and its mol­ecular conformation is stabilized *via* intra­molecular C—H⋯O and C—H⋯N contacts. The supra­molecular structure is mainly governed by C—H⋯N hydrogen-bonded centrosymmetric dimers, C—H⋯O and C—H⋯S hydrogen bonds and S⋯π and π–π stacking inter­actions which, together, lead to the formation of a layered crystal packing. The inter­molecular inter­actions were further evaluated through the mol­ecular electrostatic potential map and Hirshfeld fingerprint analysis.

## Abstract 2

The mol­ecular conformation is stabilized *via* intra­molecular C—H⋯O and C—H⋯N contacts. The supra­molecular structure is mainly governed by C—H⋯N hydrogen-bonded centrosymmetric dimers, C—H⋯O and C—H⋯S hydrogen bonds and S⋯π stacking inter­actions, which together lead to the formation of a layered crystal packing.

## Chemical context   

Compounds containing the 1,2,3-triazole scaffold are considered to be an important class of five-membered *N*-heterocycles (having two carbon and three nitro­gen atoms) because of their unique structural and chemical properties (Kolb & Sharpless, 2003 ▸; Freitas *et al.*, 2014 ▸). In the last few decades, significant attention has been paid to this kind of structural units owing to their versatile applications in the fields of materials science and medicinal chemistry (Zhou & Wang, 2012 ▸; Venugopala *et al.*, 2016 ▸). In addition, 1,2,3-triazoles have also been found to be quite relevant in objective-oriented synthesis (Billing & Nilsson, 2005 ▸), bioconjugation (Speers *et al.*, 2003 ▸) and combinatorial chemistry (Löber *et al.*, 2003 ▸). The geometrical shapes and inter­action functions of natural heterocycles and amides can be very similar to those of 1,2,3-triazoles (Thibault *et al.*, 2006 ▸).

In general, the 1,2,3-triazole nucleus is the most fundamental heterocyclic component found in various pharmacologically active agents (Agalave *et al.*, 2011 ▸). In particular, potential pharmaceuticals based on the 1,2,3-triazole ring include anti-HIV (Giffin *et al.*, 2008 ▸), anti­cancer (Singh *et al.*, 2012 ▸), anti-tubercular (Patpi *et al.*, 2012 ▸), anti­microbial (Demaray *et al.*, 2008 ▸) and anti­fungal (Lass-Floerl *et al.*, 2011 ▸) agents. This is due to the fact that the 1,2,3-triazole structural unit is stable against metabolic degradation as well as oxidation and reduction in acidic and basic conditions (Ferreira *et al.*, 2010 ▸). Importantly, this special class of structural unit is capable of forming hydrogen-bonding inter­actions (the N atom acts as an acceptor) as well as π–π stacking and other inter­molecular inter­actions with biological targets to improve their solubility (Lauria *et al.*, 2014 ▸). Hence, it is of extreme importance to explore and understand the supra­molecular structure of compounds in which the structural motif is based on 1,2,3-triazole. Keeping in mind the above-mentioned features, we report here the crystal structure and packing analysis of the title compound [5-(4-meth­oxy­phen­yl)-2-methyl-2*H*-1,2,3-tria­zol-4-yl](thio­phen-2-yl)methanone (**1**).
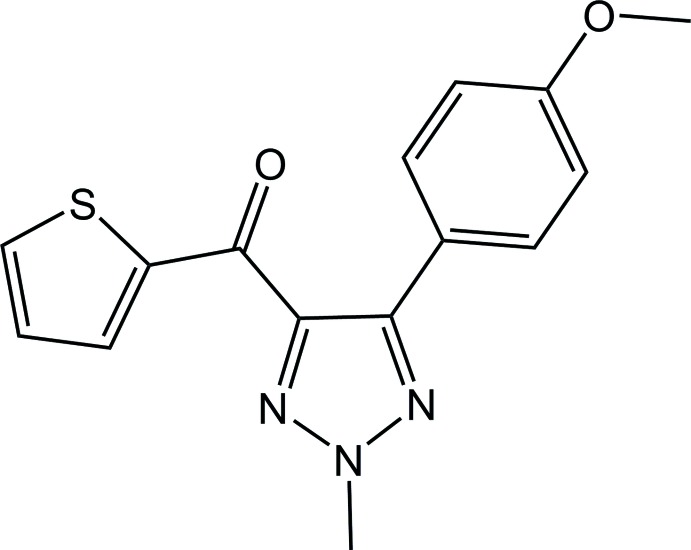


## Structural commentary   

**Figure 1:**
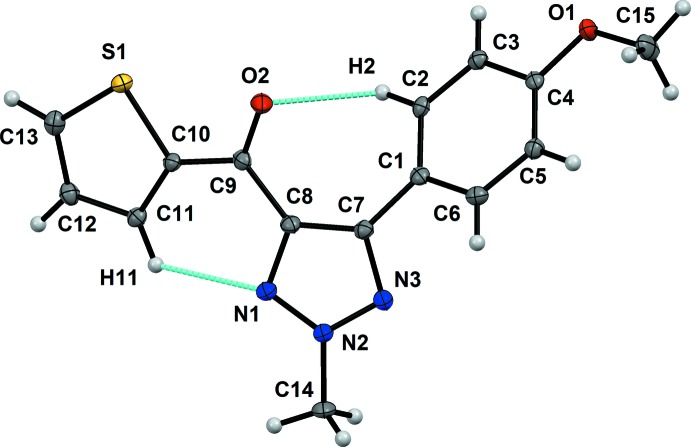
The asymmetric unit of compound **1** highlighting the intra­molecular C—H⋯O and C—H⋯N contacts. Displacement ellipsoids are drawn at the 50% probability level.

The single-crystal X-ray diffraction study shows that compound **1** crystallizes in the monoclinic space group *P*2_1_/*n* with one mol­ecule (*Z*′ = 1) in the asymmetric unit (Fig. 1 ▸). In the mol­ecular structure, the *N*-methyl­ated triazol ring is substituted at the two carbon atoms C7 and C8 by a *para*-meth­oxy phenyl and a methanone-thienyl ring, respectively, resulting in four conformationally flexible parts in the mol­ecule around the C8—C9, C9—C10, C1—C7 and C4—O1 single bonds (see Fig. 1 ▸). The conformation of the mol­ecule in the crystal is stabilized *via* intra­molecular C2—H2⋯O2 [C2⋯O2 = 2.961 (2) Å] and C11—H11⋯N1 [C11⋯N1 = 2.950 (2) Å] contacts (Fig. 1 ▸; Table 1 ▸). For this reason, the thienyl and triazole rings are nearly coplanar, with an angle of 13.63 (10)° between their mean planes, while the phenyl ring is tilted out from the mean planes of the thienyl and triazole rings by 38.84 (9) and 34.04 (10)°, respectively. It is also important to mention here that the meth­oxy group attached to C4 is in the same plane as the phenyl ring.

## Supra­molecular features   

**Figure 2:**
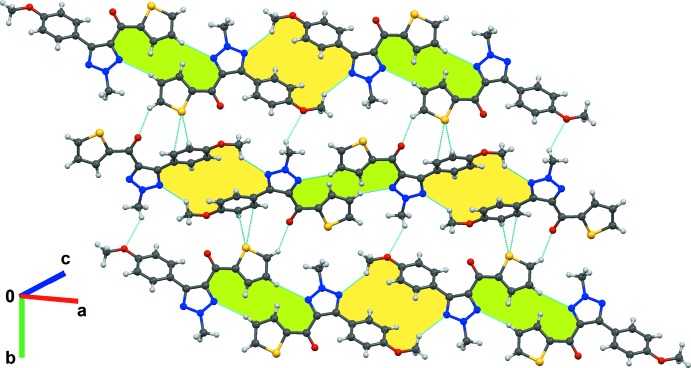
Crystal packing of **1** showing the formation of mol­ecular sheets *via* two types of centrosymmetric C—H⋯N dimers (shaded in light yellow and green), forming ribbons connected through C—H⋯O and S⋯C(π) inter­actions.

**Figure 3:**
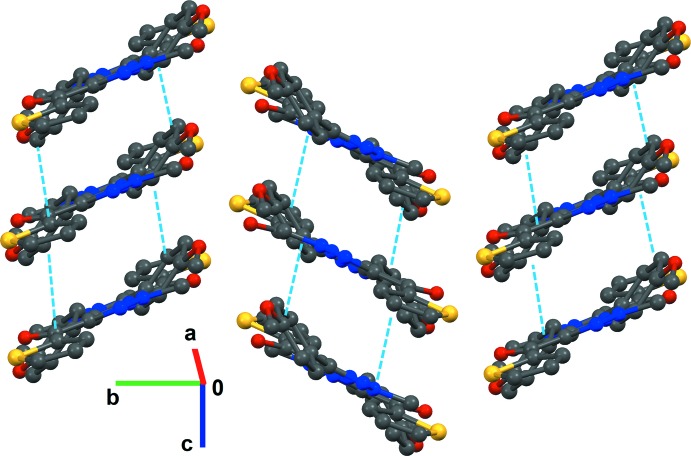
Stacking of hydrogen-bonded mol­ecular sheets *via* π–π inter­actions (dotted lines) in compound **1**. Hydrogen atoms are omitted for clarity.

In the crystal, the mol­ecules form two types of centrosymmetric, weak to very weak C—H⋯N hydrogen-bonding dimeric motifs (Table 1 ▸) involving the methyl hydrogen H15*C* (*sp* ^3^) of the meth­oxy group with the triazol nitro­gen N3 [C15⋯N3 = 3.490 (3) Å] and the thio­phene hydrogen H12 (*sp* ^2^) with the triazol nitro­gen N1 [C12⋯N1 = 3.768 (2) Å]. These are extended in an alternate fashion, forming ribbons along the [101] direction (see green and yellow shades in Fig. 2 ▸). Two such adjacent hydrogen-bonded ribbons are connected to each other *via* C*sp* ^2^/*sp* ^3^—H⋯O and S⋯C(π) [3.492 (2) Å] inter­actions along the [010] direction, forming a corrugated sheet perpendicular to the (101) plane (Fig. 2 ▸ and Table 1 ▸). These sheets are further stacked to each other by displaced π–π stacking inter­actions distances ranging from 3.375 (3) to 3.384 (4) Å through inversion and translational symmetries, and weak C3—H3⋯S1 [C3⋯S1 = 3.810 (2) Å] inter­actions (Table 1 ▸), leading to the formation of a layered packing arrangement of mol­ecules (Fig. 3 ▸).

## Analysis of mol­ecular electrostatic potential and Hirshfeld fingerprint plots   

**Figure 4:**
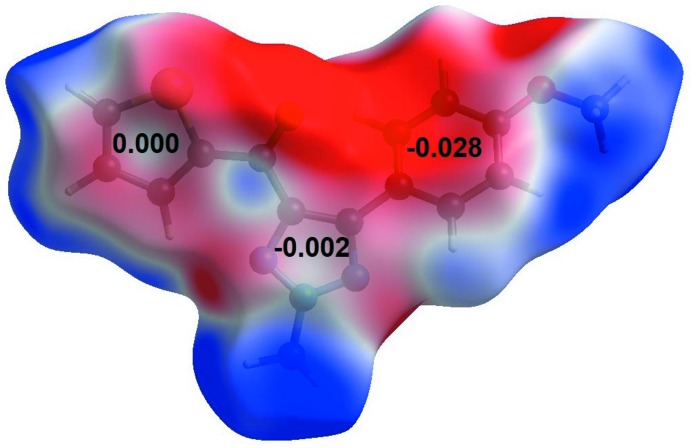
MESP of compound **1** mapped over the Hirshfeld surface with a scale of −0.03 a.u. (red) through 0.00 (white) to +0.03 a.u. (blue). The ESP values (in a.u.) for the centre of each ring are given.

**Figure 5:**
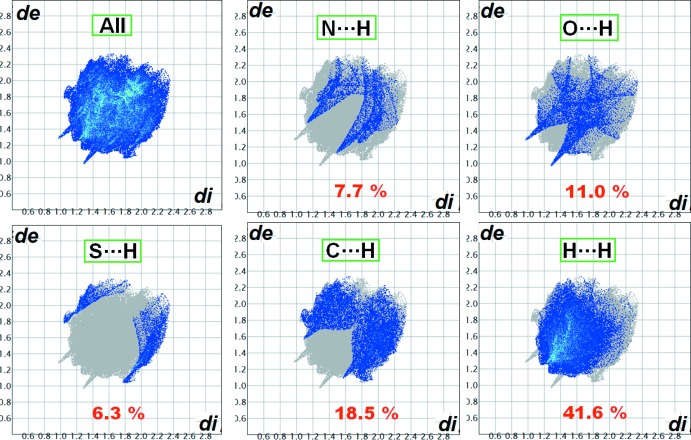
Two-dimensional full fingerprint plots and decomposed fingerprint plots over the Hirshfeld surface for various inter­molecular atom–atom contacts in compound **1**. The numbers in red indicate the percentage contributions of each contact.

A deeper insight into inter­molecular inter­actions can be obtained from mol­ecular electrostatic potential (MESP), and two-dimensional fingerprint plots (McKinnon *et al.*, 2007 ▸) mapped on the Hirshfeld surface (Spackman & Jayatilaka, 2009 ▸). All the plots were computed using the programme *CrystalExplorer 17.5* (Turner *et al.*, 2017 ▸). The MESP plot of compound **1** (Fig. 4 ▸) shows that the centres of both the triazole and thio­phene five-membered rings have nearly neutral ESP values (0.000 and −0.002 a.u., respectively), while the benzene ring is highly electronegative (−0.028 a.u.) compared to the two heterocyclic rings. This electrostatic complementarity among the rings leads to favourable stacking inter­actions in the crystal packing as a result of a layered supra­molecular architecture. Inter­molecular hydrogen-bond donors and acceptors appear as blue (positive ESP) and red (negative ESP) regions, respectively, on the surface (Fig. 4 ▸). The two-dimensional fingerprint plots and the contributions of individual inter­atomic contacts toward the overall crystal packing are shown in Fig. 5 ▸. It is observed that several directional hydrogen-bonding contacts such as N⋯H (7.7%), O⋯H (11.0%), S⋯H (6.3%) along with C⋯H (18.5%), H⋯H (41.6%) and other inter­atomic contacts stabilize the crystal packing of compound **1**.

## Database survey   

A Cambridge Structural Database (Version 5.39, update May 2018; Groom *et al.*, 2016 ▸) search for the (2-methyl-2*H*-1,2,3-triazol-4-yl)(thio­phen-2-yl)methanone subunit resulted in one hit (SONFIM; Girish *et al.*, 2014 ▸). Like compound **1**, the mol­ecular conformation of SONFIM is also stabilized by intra­molecular C—H⋯O and C—H⋯N hydrogen bonds. The supra­molecular structure of SONFIM is primarily determined by inter­molecular C—H⋯O and C—H⋯π hydrogen bonds, while C—H⋯N hydrogen bonding plays a secondary role in the overall stabilization of the crystal packing.

## Synthesis and crystallization   

The title compound was synthesized according to the procedure described elsewhere (Girish *et al.*, 2014 ▸). Single crystals of the pure compound were grown by slow evaporation of a toluene solution at room temperature (297–301 K).

## Refinement   

Crystal data, data collection and structure refinement details are given in Table 2 ▸. Hydrogen atoms were positioned geometrically and refined as riding: C—H = 0.98 Å with *U_i_*_so_(H) =1.5*U*_eq_(C) for the methyl group and C—H = 0.95Å with *U*_iso_(H) = 1.2*U*_eq_(C) for the aromatic C atoms.

## Supplementary Material

Crystal structure: contains datablock(s) I. DOI: 10.1107/S2056989018010654/xi2009sup1.cif

Structure factors: contains datablock(s) I. DOI: 10.1107/S2056989018010654/xi2009Isup2.hkl

Supporting information file. DOI: 10.1107/S2056989018010654/xi2009Isup3.cml

CCDC reference: 1850683

Additional supporting information:  crystallographic information; 3D view; checkCIF report

## Figures and Tables

**Table 1:** Hydrogen-bond geometry (Å, °)

*D*—H⋯*A*	*D*—H	H⋯*A*	*D*⋯*A*	*D*—H⋯*A*
C11—H11⋯N1	0.95	2.41	2.950 (2)	116
C2—H2⋯O2	0.95	2.42	2.961 (2)	113
C3—H3⋯S1^i^	0.95	2.96	3.810 (2)	149
C15—H15*A*⋯O2^ii^	0.98	2.98	3.828 (3)	146
C15—H15*C*⋯N3^iii^	0.98	2.73	3.490 (3)	135
C12—H12⋯N1^iv^	0.95	2.95	3.768 (2)	145
C13—H13⋯O2^v^	0.95	2.38	3.191 (2)	143
C14—H14*C*⋯O1^vi^	0.98	2.67	3.230 (2)	117

Symmetry codes: (i) 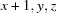; (ii) 
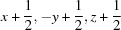
; (iii) 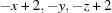; (iv) 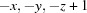; (v) 
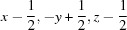
; (vi) 
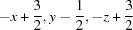
.

**Table 2:** Experimental details

Crystal data	
Chemical formula	C_15_H_13_N_3_O_2_S
*M*_r_	299.34
Crystal system, space group	Monoclinic, *P*2_1_/*n*
Temperature (K)	100
*a*, *b*, *c* (Å)	8.5851 (10), 16.8986 (19), 9.3455 (11)
β (°)	92.465 (4)
*V* (Å^3^)	1354.6 (3)
*Z*	4
Radiation type	Mo *K*α
μ (mm^−1^)	0.25
Crystal size (mm)	0.30 × 0.10 × 0.06

Data collection	
Diffractometer	Bruker APEXII D8 Venture CMOS
Absorption correction	Multi-scan (*SADABS*; Bruker, 2012 ▸)
*T*_min_, *T*_max_	0.619, 0.746
No. of measured, independent and observed [*I* > 2σ(*I*)] reflections	17149, 3962, 2914
*R*_int_	0.065
(sin θ/λ)_max_ (Å^−1^)	0.705

Refinement	
*R*[*F* ^2^ > 2σ(*F* ^2^)], *wR*(*F* ^2^), *S*	0.053, 0.114, 1.03
No. of reflections	3962
No. of parameters	192
H-atom treatment	H-atom parameters constrained
Δρ_max_, Δρ_min_ (e Å^−3^)	0.46, −0.53

Computer programs: *APEX2* and *SAINT* (Bruker, 2012 ▸), *SIR2014* (Burla *et al.*, 2015 ▸), *SHELXL2018* (Sheldrick, 2015 ▸), *Mercury* (Macrae *et al.*, 2006 ▸), *WinGX* (Farrugia, 2012 ▸) and *PLATON* (Spek, 2009 ▸).

## supplementary crystallographic information

### Crystal data

**Table:**

C_15_H_13_N_3_O_2_S	*F*(000) = 624
*M**_r_* = 299.34	*D*_x_ = 1.468 Mg m^−^^3^
Monoclinic, *P*2_1_/*n*	Mo *K*α radiation, λ = 0.71073 Å
*a* = 8.5851 (10) Å	Cell parameters from 6642 reflections
*b* = 16.8986 (19) Å	θ = 2.4–30.0°
*c* = 9.3455 (11) Å	µ = 0.25 mm^−^^1^
β = 92.465 (4)°	*T* = 100 K
*V* = 1354.6 (3) Å^3^	Plate, yellow
*Z* = 4	0.30 × 0.10 × 0.06 mm

### Data collection

**Table:**

Bruker APEXII D8 Venture CMOS diffractometer	2914 reflections with *I* > 2σ(*I*)
φ and ω scans	*R*_int_ = 0.065
Absorption correction: multi-scan (SADABS; Bruker, 2012)	θ_max_ = 30.1°, θ_min_ = 2.4°
*T*_min_ = 0.619, *T*_max_ = 0.746	*h* = −12→11
17149 measured reflections	*k* = −23→23
3962 independent reflections	*l* = −10→13

### Refinement

**Table:**

Refinement on *F*^2^	0 restraints
Least-squares matrix: full	Hydrogen site location: inferred from neighbouring sites
*R*[*F*^2^ > 2σ(*F*^2^)] = 0.053	H-atom parameters constrained
*wR*(*F*^2^) = 0.114	*w* = 1/[σ^2^(*F*_o_^2^) + (0.037*P*)^2^ + 1.3716*P*] where *P* = (*F*_o_^2^ + 2*F*_c_^2^)/3
*S* = 1.03	(Δ/σ)_max_ < 0.001
3962 reflections	Δρ_max_ = 0.46 e Å^−^^3^
192 parameters	Δρ_min_ = −0.53 e Å^−^^3^

### Special details

**Table:**

Geometry. All esds (except the esd in the dihedral angle between two l.s. planes) are estimated using the full covariance matrix. The cell esds are taken into account individually in the estimation of esds in distances, angles and torsion angles; correlations between esds in cell parameters are only used when they are defined by crystal symmetry. An approximate (isotropic) treatment of cell esds is used for estimating esds involving l.s. planes.

### Fractional atomic coordinates and isotropic or equivalent isotropic displacement parameters (Å^2^)

**Table:**

	*x*	*y*	*z*	*U*_iso_*/*U*_eq_	
S1	0.22992 (6)	0.20914 (3)	0.39316 (5)	0.01627 (13)	
O1	1.18053 (15)	0.18243 (8)	0.81322 (14)	0.0166 (3)	
O2	0.48083 (16)	0.18611 (8)	0.60466 (16)	0.0200 (3)	
N1	0.35646 (18)	−0.00987 (9)	0.68224 (18)	0.0160 (3)	
N3	0.57606 (18)	−0.03357 (10)	0.81538 (18)	0.0158 (3)	
N2	0.43569 (18)	−0.05846 (9)	0.76814 (18)	0.0162 (3)	
C1	0.7388 (2)	0.08137 (10)	0.7775 (2)	0.0130 (4)	
C11	0.1653 (2)	0.06385 (11)	0.4486 (2)	0.0137 (4)	
H11	0.167530	0.012226	0.488904	0.016*	
C4	1.0365 (2)	0.14778 (10)	0.8101 (2)	0.0130 (4)	
C3	0.9476 (2)	0.15862 (11)	0.6832 (2)	0.0132 (4)	
H3	0.988464	0.188552	0.607394	0.016*	
C7	0.5901 (2)	0.03848 (11)	0.7557 (2)	0.0132 (4)	
C5	0.9775 (2)	0.10385 (11)	0.9210 (2)	0.0150 (4)	
H5	1.037167	0.096550	1.007982	0.018*	
C10	0.2700 (2)	0.12300 (11)	0.4876 (2)	0.0128 (4)	
C6	0.8294 (2)	0.07061 (11)	0.9028 (2)	0.0147 (4)	
H6	0.789370	0.039832	0.977967	0.018*	
C2	0.8005 (2)	0.12602 (11)	0.6675 (2)	0.0131 (4)	
H2	0.740456	0.134022	0.580881	0.016*	
C8	0.4519 (2)	0.05345 (11)	0.6724 (2)	0.0138 (4)	
C9	0.4044 (2)	0.12489 (11)	0.5897 (2)	0.0141 (4)	
C15	1.2758 (2)	0.17414 (14)	0.9413 (2)	0.0245 (5)	
H15A	1.224395	0.199444	1.020945	0.037*	
H15B	1.376982	0.199448	0.928427	0.037*	
H15C	1.291563	0.117828	0.962413	0.037*	
C12	0.0541 (2)	0.08913 (12)	0.3416 (2)	0.0160 (4)	
H12	−0.026154	0.056184	0.301494	0.019*	
C13	0.0753 (2)	0.16613 (12)	0.3025 (2)	0.0173 (4)	
H13	0.011053	0.192791	0.232674	0.021*	
C14	0.3821 (2)	−0.13873 (11)	0.7969 (2)	0.0213 (4)	
H14A	0.273221	−0.144368	0.762375	0.032*	
H14B	0.390158	−0.148830	0.900188	0.032*	
H14C	0.446872	−0.176823	0.747341	0.032*	

### Atomic displacement parameters (Å^2^)

**Table:**

	*U*^11^	*U*^22^	*U*^33^	*U*^12^	*U*^13^	*U*^23^
S1	0.0164 (2)	0.0123 (2)	0.0201 (3)	−0.00008 (18)	0.00098 (18)	0.00335 (19)
O1	0.0124 (6)	0.0188 (7)	0.0183 (7)	−0.0030 (5)	−0.0029 (5)	0.0017 (6)
O2	0.0164 (7)	0.0107 (6)	0.0325 (8)	−0.0021 (5)	−0.0028 (6)	0.0009 (6)
N1	0.0151 (8)	0.0118 (7)	0.0209 (9)	0.0003 (6)	−0.0009 (7)	0.0019 (7)
N3	0.0124 (8)	0.0154 (8)	0.0195 (8)	−0.0002 (6)	−0.0003 (6)	0.0005 (7)
N2	0.0135 (8)	0.0125 (8)	0.0223 (9)	−0.0006 (6)	−0.0013 (7)	0.0036 (7)
C1	0.0136 (9)	0.0092 (8)	0.0162 (10)	0.0008 (7)	0.0014 (7)	−0.0018 (7)
C11	0.0150 (9)	0.0120 (8)	0.0143 (9)	0.0017 (7)	0.0010 (7)	−0.0010 (7)
C4	0.0123 (8)	0.0094 (8)	0.0173 (9)	0.0006 (7)	0.0001 (7)	−0.0019 (7)
C3	0.0143 (9)	0.0117 (8)	0.0136 (9)	0.0019 (7)	0.0019 (7)	−0.0002 (7)
C7	0.0139 (9)	0.0110 (8)	0.0148 (9)	0.0012 (7)	0.0020 (7)	−0.0002 (7)
C5	0.0143 (9)	0.0150 (9)	0.0154 (9)	0.0009 (7)	−0.0012 (7)	0.0002 (7)
C10	0.0121 (9)	0.0105 (8)	0.0160 (9)	0.0017 (7)	0.0028 (7)	0.0008 (7)
C6	0.0173 (9)	0.0123 (9)	0.0145 (9)	−0.0005 (7)	0.0022 (7)	0.0008 (7)
C2	0.0132 (9)	0.0124 (8)	0.0136 (9)	0.0032 (7)	−0.0004 (7)	−0.0010 (7)
C8	0.0138 (9)	0.0099 (8)	0.0178 (10)	−0.0005 (7)	0.0009 (7)	−0.0010 (7)
C9	0.0115 (9)	0.0116 (8)	0.0193 (10)	0.0012 (7)	0.0028 (7)	−0.0015 (7)
C15	0.0189 (10)	0.0338 (12)	0.0203 (11)	−0.0080 (9)	−0.0066 (8)	0.0034 (9)
C12	0.0144 (9)	0.0170 (9)	0.0164 (10)	−0.0014 (7)	−0.0015 (7)	−0.0028 (8)
C13	0.0160 (9)	0.0204 (10)	0.0156 (10)	0.0025 (7)	0.0000 (7)	−0.0005 (8)
C14	0.0199 (10)	0.0116 (9)	0.0322 (12)	−0.0030 (8)	−0.0010 (9)	0.0070 (8)

### Geometric parameters (Å, º)

**Table:**

S1—C13	1.706 (2)	C3—C2	1.380 (3)
S1—C10	1.7292 (19)	C3—H3	0.9500
O1—C4	1.368 (2)	C7—C8	1.413 (3)
O1—C15	1.427 (2)	C5—C6	1.394 (3)
O2—C9	1.230 (2)	C5—H5	0.9500
N1—N2	1.317 (2)	C10—C9	1.466 (3)
N1—C8	1.353 (2)	C6—H6	0.9500
N3—N2	1.334 (2)	C2—H2	0.9500
N3—C7	1.347 (2)	C8—C9	1.481 (3)
N2—C14	1.461 (2)	C15—H15A	0.9800
C1—C6	1.390 (3)	C15—H15B	0.9800
C1—C2	1.398 (3)	C15—H15C	0.9800
C1—C7	1.475 (3)	C12—C13	1.365 (3)
C11—C10	1.383 (3)	C12—H12	0.9500
C11—C12	1.419 (3)	C13—H13	0.9500
C11—H11	0.9500	C14—H14A	0.9800
C4—C5	1.388 (3)	C14—H14B	0.9800
C4—C3	1.394 (3)	C14—H14C	0.9800
			
C13—S1—C10	91.62 (9)	C1—C6—H6	119.2
C4—O1—C15	117.45 (15)	C5—C6—H6	119.2
N2—N1—C8	103.66 (15)	C3—C2—C1	120.72 (17)
N2—N3—C7	104.09 (15)	C3—C2—H2	119.6
N1—N2—N3	116.16 (15)	C1—C2—H2	119.6
N1—N2—C14	122.16 (16)	N1—C8—C7	108.49 (16)
N3—N2—C14	121.29 (16)	N1—C8—C9	121.73 (16)
C6—C1—C2	118.34 (17)	C7—C8—C9	129.73 (17)
C6—C1—C7	120.11 (17)	O2—C9—C10	119.57 (17)
C2—C1—C7	121.13 (17)	O2—C9—C8	119.53 (17)
C10—C11—C12	112.23 (17)	C10—C9—C8	120.88 (16)
C10—C11—H11	123.9	O1—C15—H15A	109.5
C12—C11—H11	123.9	O1—C15—H15B	109.5
O1—C4—C5	124.87 (17)	H15A—C15—H15B	109.5
O1—C4—C3	115.02 (17)	O1—C15—H15C	109.5
C5—C4—C3	120.10 (17)	H15A—C15—H15C	109.5
C2—C3—C4	120.21 (18)	H15B—C15—H15C	109.5
C2—C3—H3	119.9	C13—C12—C11	112.45 (17)
C4—C3—H3	119.9	C13—C12—H12	123.8
N3—C7—C8	107.60 (16)	C11—C12—H12	123.8
N3—C7—C1	118.64 (16)	C12—C13—S1	112.53 (15)
C8—C7—C1	133.56 (17)	C12—C13—H13	123.7
C4—C5—C6	118.99 (17)	S1—C13—H13	123.7
C4—C5—H5	120.5	N2—C14—H14A	109.5
C6—C5—H5	120.5	N2—C14—H14B	109.5
C11—C10—C9	132.22 (17)	H14A—C14—H14B	109.5
C11—C10—S1	111.18 (14)	N2—C14—H14C	109.5
C9—C10—S1	116.60 (13)	H14A—C14—H14C	109.5
C1—C6—C5	121.63 (18)	H14B—C14—H14C	109.5
			
C8—N1—N2—N3	−0.6 (2)	C4—C5—C6—C1	−1.0 (3)
C8—N1—N2—C14	−173.55 (18)	C4—C3—C2—C1	−0.4 (3)
C7—N3—N2—N1	0.5 (2)	C6—C1—C2—C3	−0.1 (3)
C7—N3—N2—C14	173.46 (18)	C7—C1—C2—C3	−172.69 (17)
C15—O1—C4—C5	−1.4 (3)	N2—N1—C8—C7	0.5 (2)
C15—O1—C4—C3	179.19 (17)	N2—N1—C8—C9	−177.17 (17)
O1—C4—C3—C2	179.69 (16)	N3—C7—C8—N1	−0.3 (2)
C5—C4—C3—C2	0.2 (3)	C1—C7—C8—N1	174.3 (2)
N2—N3—C7—C8	−0.1 (2)	N3—C7—C8—C9	177.16 (19)
N2—N3—C7—C1	−175.62 (16)	C1—C7—C8—C9	−8.3 (4)
C6—C1—C7—N3	−30.9 (3)	C11—C10—C9—O2	179.7 (2)
C2—C1—C7—N3	141.55 (18)	S1—C10—C9—O2	0.5 (2)
C6—C1—C7—C8	155.0 (2)	C11—C10—C9—C8	1.3 (3)
C2—C1—C7—C8	−32.5 (3)	S1—C10—C9—C8	−177.88 (14)
O1—C4—C5—C6	−178.95 (17)	N1—C8—C9—O2	166.57 (18)
C3—C4—C5—C6	0.4 (3)	C7—C8—C9—O2	−10.5 (3)
C12—C11—C10—C9	−178.84 (19)	N1—C8—C9—C10	−15.0 (3)
C12—C11—C10—S1	0.4 (2)	C7—C8—C9—C10	167.85 (19)
C13—S1—C10—C11	−0.13 (15)	C10—C11—C12—C13	−0.5 (2)
C13—S1—C10—C9	179.21 (15)	C11—C12—C13—S1	0.4 (2)
C2—C1—C6—C5	0.8 (3)	C10—S1—C13—C12	−0.14 (16)
C7—C1—C6—C5	173.47 (17)		

### Hydrogen-bond geometry (Å, º)

**Table:**

*D*—H···*A*	*D*—H	H···*A*	*D*···*A*	*D*—H···*A*
C11—H11···N1	0.95	2.41	2.950 (2)	116
C2—H2···O2	0.95	2.42	2.961 (2)	113
C3—H3···S1^i^	0.95	2.96	3.810 (2)	149
C15—H15*A*···O2^ii^	0.98	2.98	3.828 (3)	146
C15—H15*C*···N3^iii^	0.98	2.73	3.490 (3)	135
C12—H12···N1^iv^	0.95	2.95	3.768 (2)	145
C13—H13···O2^v^	0.95	2.38	3.191 (2)	143
C14—H14*C*···O1^vi^	0.98	2.67	3.230 (2)	117

Symmetry codes: (i) *x*+1, *y*, *z*; (ii) *x*+1/2, −*y*+1/2, *z*+1/2; (iii) −*x*+2, −*y*, −*z*+2; (iv) −*x*, −*y*, −*z*+1; (v) *x*−1/2, −*y*+1/2, *z*−1/2; (vi) −*x*+3/2, *y*−1/2, −*z*+3/2.
